# Design and Development of a Precision Spraying Control System for Orchards Based on Machine Vision Detection

**DOI:** 10.3390/s25123799

**Published:** 2025-06-18

**Authors:** Yu Luo, Xiaoli He, Hanwen Shi, Simon X. Yang, Lepeng Song, Ping Li

**Affiliations:** 1 School of Electronic and Electrical Engineering, Chongqing University of Science & Technology, Chongqing 401331, China; 2005044@cqust.edu.cn (Y.L.); 2023204018@cqust.edu.cn (X.H.); 2024204014@cqust.edu.cn (H.S.); 2 Advanced Robotics and Intelligent Systems Laboratory, School of Engineering, University of Guelph, Guelph, ON N1G 2W1, Canada; syang@uoguelph.ca; 3 Chongqing Academy of Agricultural Sciences, Chongqing 400039, China; lipinghanxe2005@126.com

**Keywords:** precision spraying, machine vision, pesticide spray volume, ESO fuzzy adaptive control

## Abstract

Precision spraying technology has attracted increasing attention in orchard production management. Traditional chemical pesticide application relies on subjective judgment, leading to fluctuations in pesticide usage, low application efficiency, and environmental pollution. This study proposes a machine vision-based precision spraying control system for orchards. First, a canopy leaf wall area calculation method was developed based on a multi-iteration GrabCut image segmentation algorithm, and a spray volume calculation model was established. Next, a fuzzy adaptive control algorithm based on an extended state observer (ESO) was proposed, along with the design of flow and pressure controllers. Finally, the precision spraying system’s performance tests were conducted in laboratory and field environments. The indoor experiments consisted of three test sets, each involving six citrus trees, totaling eighteen trees arranged in two staggered rows, with an interrow spacing of 3.4 m and an intra-row spacing of 2.5 m; the nozzle was positioned approximately 1.3 m from the canopy surface. Similarly, the field experiments included three test sets, each selecting eight citrus trees, totaling twenty-four trees, with an average height of approximately 1.5 m and a row spacing of 3 m, representing a typical orchard environment for performance validation. Experimental results demonstrated that the system reduced spray volume by 59.73% compared to continuous spraying, by 30.24% compared to PID control, and by 19.19% compared to traditional fuzzy control; meanwhile, the pesticide utilization efficiency increased by 61.42%, 26.8%, and 19.54%, respectively. The findings of this study provide a novel technical approach to improving agricultural production efficiency, enhancing fruit quality, reducing pesticide use, and promoting environmental protection, demonstrating significant application value.

## 1. Introduction

In modern orchard management, chemical pesticide spraying remains one of the key measures for controlling pests and diseases, widely adopted due to its ease of operation, low cost, and rapid effectiveness [1,2]. However, traditional spraying methods often rely on the experience of the operator and lack precision, leading to pesticide waste, inconsistent pest control efficacy, and increased risks of environmental pollution and pesticide residues [3,4]. As Toolkiattiwong et al. highlight, excessive agricultural inputs—particularly fertilizers, herbicides, and insecticides—can significantly elevate carbon footprints, water footprints, human health risks, and freshwater ecotoxicity [5]. Consequently, the development of precision spraying technologies has become a crucial pathway for promoting green and efficient transformation in agriculture.

Precise detection of fruit tree canopy structural parameters is critical in improving pesticide utilization efficiency in orchards. As an essential prerequisite for precision spraying, the reliable quantification of canopy characteristics places higher demands on sensing technologies. Traditional photoelectric and ultrasonic sensors are easily affected by environmental interference. Although LiDAR offers high accuracy, its high cost and the complexity of point cloud processing limit its widespread application. In contrast, machine vision technology, with its advantages of high resolution, real-time processing capability, and lower hardware cost, is gradually becoming an ideal perception method in orchard-spraying systems [6]. Significant progress has been made in applying machine vision for orchard canopy detection in recent years. Jin et al. applied an FCN model combined with the STDC backbone network to segment apple canopy size, achieving better results compared to the PP-LiteSeg model [7]; Zhu et al. proposed an improved YOLOv4-based method for fruit tree canopy detection, effectively overcoming noise in orchard environments while achieving rapid and accurate canopy recognition with a lightweight model [8]; Neupane et al. utilized YOLOv8m for canopy detection and mango counting, obtaining an accuracy comparable to the Mask R-CNN model but with a relatively faster detection speed [9]; Xu et al. employed ECA-Unet combined with multispectral images for canopy segmentation, which outputs segmentation results more quickly and effectively compared to traditional semi-automatic crown segmentation methods [10]. Although existing studies have achieved remarkable results, most methods rely on deep convolutional networks, which have high computational complexity, limited ability to handle blurred boundary regions, and difficulties adapting to orchard scenes with severe illumination variations. Therefore, this study adopts the GrabCut algorithm for fruit tree canopy segmentation. This algorithm possesses advantages such as strong edge preservation, high background robustness, and moderate computational efficiency, making it suitable for real-time canopy extraction in natural environments and effectively compensating for the shortcomings of deep models in lightweight deployment and boundary detail processing.

Based on the precise detection of fruit tree canopies, effective acquisition of actual pesticide application amounts and precise control of spraying volumes are key to improving the pesticide utilization efficiency [11]. Precision spraying control technology has achieved significant domestic and international research results, especially in applying PID control, fuzzy control, and intelligent control. The primary focus is improving the spraying accuracy while reducing pesticide waste and environmental pollution [12]. Zangina et al. used a model-predictive controller to address how to determine the correct timing, quantity, and location of pesticide application in agriculture [13]. Zhang et al. employed a precision variable plant protection drone equipped with an ultrasonic sensor array for spraying, reducing pesticide usage by 34.5% [14]. Precision spraying primarily involves controlling electromagnetic valves via pulse-width modulation (PWM). Han et al. significantly reduced the holding current of electromagnetic valves by varying the frequency and duty cycle of PWM signals, saving 92% of energy and effectively lowering surface temperature [15]. Wen et al. applied PID and PWM controllers combined with sensor technology to interpret real-time prescription maps for precision spraying, maintaining flow deviation within 2.16% [16]. Schutz et al. utilized a fuzzy gain-scheduled advanced embedded generalized predictive controller for agricultural sprayers, where adding a fuzzy logic system significantly improved the spraying accuracy of nonlinear agricultural sprayers [17]. However, these methods commonly face challenges such as nonlinearity, time variance, and significant delays [18]. To resolve the conflict between spraying rapidity and system stability, particularly to significantly reduce pesticide waste, relying solely on traditional PID or fuzzy control methods is insufficient. The ESO fuzzy adaptive control method can overcome various disturbances while achieving precise spraying control.

## 2. Materials and Methods

**Figure 1:**
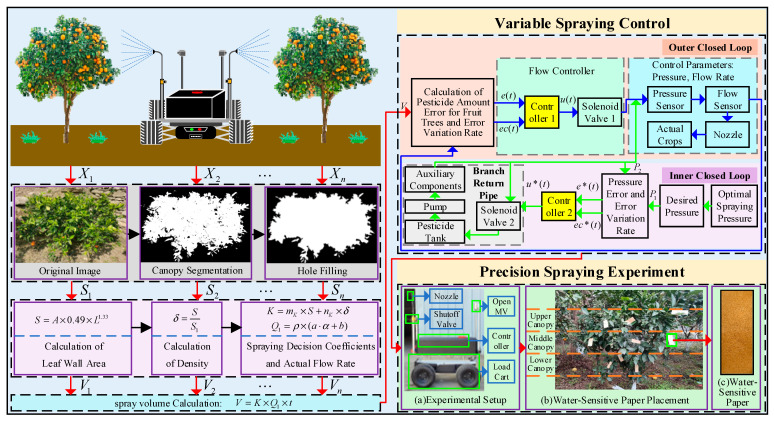
Overall design plan of the precision spraying system.

This paper proposes a machine vision-based ESO fuzzy adaptive control algorithm. ESO (extended state observer) is a state-estimation technique designed to simultaneously estimate the system states and total disturbances, including external disturbances and model uncertainties [19]. The research objectives are as follows: (1) to detect fruit tree canopy feature parameters using machine vision and construct a spray volume calculation model based on canopy leaf wall area and density; (2) to develop a precision spraying control system based on ESO fuzzy adaptive control, which regulates pesticide flow and pressure through PWM-controlled solenoid valves, and to validate the system via MATLAB2019b (version 9.7.0) simulations; (3) to implement a prototype precision spraying system through the integrated development of OpenMV and STM32, enabling detection of canopy feature parameters, accurate spray volume calculation, and precise control of spraying flow and pressure. The overall design scheme of this study is illustrated in Figure 1.

### 2.1. Research on Tree Canopy Detection Method Based on Machine Vision and Pesticide Spray Volume Model Design

#### 2.1.1. Tree Canopy Image Segmentation Based on GrabCut Algorithm

**Figure 2:**
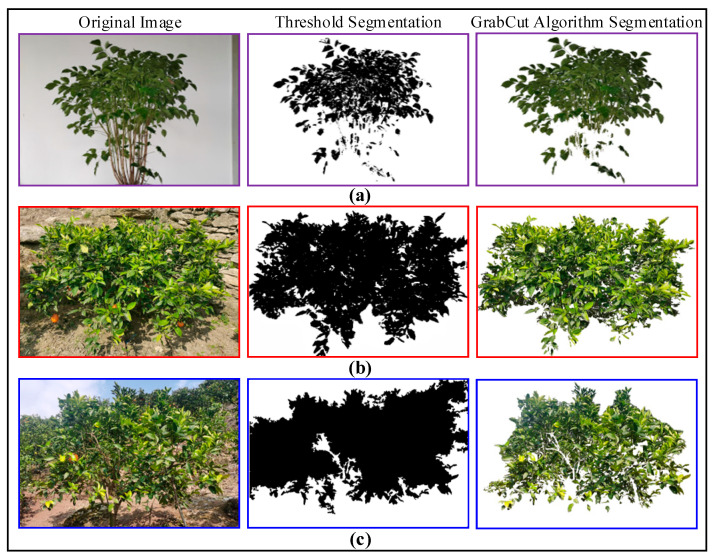
Results of citrus tree canopy image segmentation.

In tree canopy image segmentation, precise separation is challenging due to factors such as differences in sunlight reflection characteristics, shadows, exposure, and complex backgrounds in various parts of the canopy. After comparing various algorithms, this paper selects the GrabCut algorithm [20] for the segmentation of different objects, with the results shown in Figure 2.

Table 1 compares the segmentation performance of GrabCut and the thresholding method on fruit tree canopy images.

Figure 2a illustrates a comparison of segmentation results between thresholding and the GrabCut algorithm under a uniform background. Figure 2b illustrates a comparison of segmentation results between thresholding and the GrabCut algorithm under a complex ground background. Figure 2c illustrates a comparison of segmentation results between thresholding and the GrabCut algorithm under a multi-tree background.

The table compares parameter settings and performance metrics between the GrabCut algorithm and the thresholding method applied to fruit tree canopy image segmentation. The results demonstrate that, with appropriately configured iteration counts and energy function weights, GrabCut significantly outperforms the thresholding method in metrics such as Intersection over Union (IoU), precision, and recall, highlighting its superiority in handling complex canopy segmentation tasks.

#### 2.1.2. Tree Canopy Feature Parameter Detection Method Based on Machine Vision

**Figure 3:**
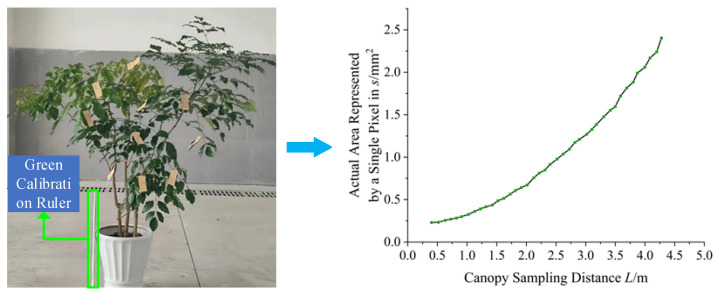
Experiment to determine the relationship between area represented by a single pixel and measurement distance.

To convert image pixels into actual dimensions, a 600 mm × 100 mm green calibration ruler was introduced as a reference, placed in the same plane as the tree canopy. Image processing was used to extract the pixel ratio of the canopy and the calibration ruler. To establish a mapping relationship between pixels and actual area, a sampling experiment was designed: starting from 0.6 m, the distance was increased by 0.1 m each time, and 37 consecutive images were captured, recording the pixel values of the calibration ruler at each distance, as shown in Figure 3.

Data fitting was performed using MATLAB to establish a conversion relationship between pixels and actual area. The relationship is given by Equation (1).


(1) $$s=0.49\times L^{1.33}$$

In the equation, *s* represents the actual area corresponding to a single pixel, and *L* is the sampling distance of the canopy image.

Next, tree canopy leaf wall area detection is performed. Image preprocessing is applied to enhance processing effectiveness, followed by image segmentation to distinguish between the leaves and the background. After setting a threshold, pixel points are identified and counted to accurately calculate the actual leaf wall area, as shown in Equation (2).


(2) S=A×s

In the equation, *S* represents the actual leaf wall area of the canopy, *A* is the number of canopy leaf pixels, and *s* is the actual area represented by one pixel, as calculated in the previous equation.

**Figure 4:**
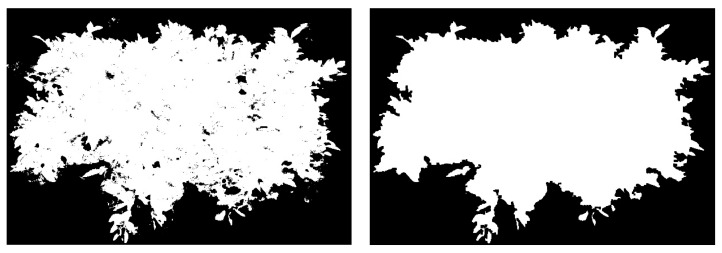
Canopy image before and after filling.

Finally, tree canopy density detection is performed. The detection steps are as follows: (1) Capture a frontal image of the canopy; (2) perform image preprocessing and segmentation; (3) set parameters and thresholds, extract the canopy leaf region through image analysis, fit the canopy contour, and use morphological techniques to fill the holes within the contour. The filled canopy image is shown in Figure 4, and the contour area is calculated; (4) compare the canopy contour area with the leaf wall area to obtain the density calculation equation.


(3) $$\delta =\frac{S}{S_{1}}$$

Equation (3) represents the canopy density; *S*_1_ is the contour area of the canopy after filling, and *S* is the leaf wall area of the canopy.

#### 2.1.3. Pesticide Spray Volume Calculation Model Based on Canopy Feature Parameters

Both the spray flow coefficient and the spray volume adjustment coefficient are dimensionless. The former represents the flow output capability of the nozzle under a given unit pressure. A nozzle flow calibration experiment is conducted to determine this coefficient by adjusting the PWM duty cycle, spray flow, and PWM controller settings [21]. During the experiment, readings from the flow meter and pressure gauge are recorded at various levels, and the coefficient is calculated from these measurements.

The latter coefficient reflects the ratio between the actual and theoretical spray volumes. When the actual volume exceeds the theoretical one, the adjustment coefficient is greater than 1; conversely, it is less than 1. The experiment involves calculating the theoretical spray volume, adjusting the spray pump to the predetermined pressure within a controlled environment, and simultaneously measuring the flow rate, time, and actual liquid volume in a standard container. The adjustment coefficient is then calculated by comparing the actual and theoretical spray volumes, as shown in Equation (4).


(4) $$\rho =\frac{V_{r}}{V_{t}}$$

In the equation, *V_r_* represents the theoretical spray volume, while *V_t_* represents the actual spray volume.

In this study, the PWM signal output from the spray controller is used to control the solenoid valve opening, enabling variable spraying. The larger the PWM duty cycle, the greater the valve opening, leading to a higher application rate; conversely, a more minor duty cycle results in a lower application rate. The relationship between the nozzle’s actual flow rate and the pulse signal duty cycle is expressed in Equation (5).


(5) $$Q_{1}=\rho \times (a\cdot \alpha +b)$$

In the equation, *Q*_1_ represents the actual flow rate of the nozzle, and *a* and *b* are the flow coefficients determined by the nozzle flow model, with the calibrated values of *a* and *b* being 0.867 and 0.219, respectively. *ρ* is the spray volume adjustment coefficient, with an experimental value of 0.832, and *α* denotes the duty cycle of the pulse signal.

The application rate is determined by both the canopy density and leaf wall area; i.e., *K* is weighted by two characteristic parameters, and its calculation model is shown in Equation (6).


(6) $$K=m_{K}\times S+n_{K}\times \delta$$

In the equation, *S* represents the canopy leaf wall area, δ is the canopy density, and *m_K_* and *n_K_* are the weight coefficients. *m_K_* is the ratio of the actual leaf wall area of the tree under measurement to the average leaf wall area of the trees in the orchard. At the same time, *n_K_* is the ratio of the actual canopy density of the tree under measurement to the average canopy density of the trees in the orchard.

The pesticide application rate calculation model is used in the decision-making process for variable-rate spraying, to calculate the required pesticide volume in real time. This model computes the decision coefficient based on the detected canopy leaf wall area and density and, combined with the time taken for the unmanned plant protection machine to pass over the trees, determines the pesticide application rate.


(7) $$V=K\times Q_{1}\times t=\rho \times K\times t(a\times \alpha +b)$$

In the equation, *V* represents the pesticide application rate for the tree, *t* is the application time, experimentally determined to be 1.5 s, and *K* is the pesticide application decision coefficient.

### 2.2. Design of a Precision Spraying Control Algorithm

**Figure 5:**
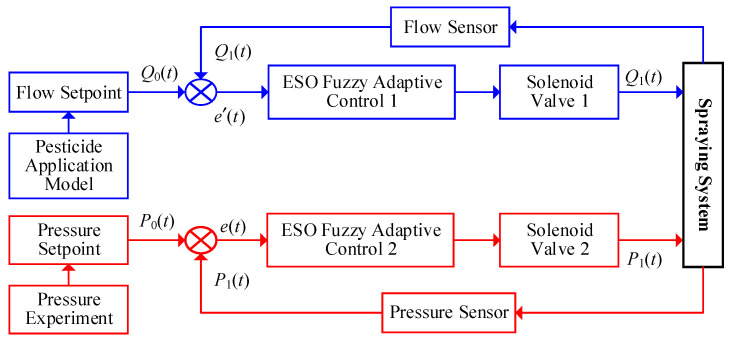
Variable spraying structure with dual closed-loop control.

Traditional single-control methods are challenging to meet the demands of precision pesticide application. To address this, this study proposes a dual closed-loop control scheme (as shown in Figure 5), which includes two independent control loops for flow rate and pressure [22]. The outer loop (blue) dynamically adjusts the pesticide flow rate based on real-time data from the flow sensor, ensuring accurate delivery. The inner loop (red) dynamically regulates the spray pressure based on real-time data from the pressure sensor, optimizing droplet deposition efficiency. This scheme improves the application accuracy and droplet deposition, enhances pesticide adherence, and increases both application efficiency and control effectiveness.

Note: *Q*_0_(*t*) is the required pesticide application rate for the tree (determined through the analysis of the tree canopy characteristic parameters detected above), *Q*_1_(*t*) is the measured pesticide application rate from the flow sensor, and $e^{\prime }(t)$ = *Q*_0_(*t*) − *Q*_1_(*t*) is the difference between the required pesticide application rate and the measured pesticide application rate from the flow sensor, referred to as the application rate deviation. $(de/dt{)}^{\prime }=de^{\prime }(t)/dt$ represents the rate of change in the application rate deviation. *P*_0_(*t*) is the required pressure in the main pipeline (calculated from the droplet size detected by the industrial camera), *P*_1_(*t*) is the pressure value measured by the pressure sensor, and *e*(*t*) = *P*_0_(*t*) − *P*_1_(*t*) is the difference between the required pressure in the main pipeline and the pressure value measured by the pressure sensor.

The pesticide application system acquires the canopy characteristic parameters of the tree through the camera and calculates the required pesticide application rate, *Q*_0_(*t*), as the input. The difference between the spray volume measured by the flow sensor and the actual spray volume is used to obtain the deviation in spray volume and its rate of change, $e^{\prime }(t)$ and $(de/dt{)}^{\prime }$, which are used as inputs to the controller. The output from the controller actuates the solenoid valve to control the flow rate and achieve precise pesticide application. Pressure control employs the same dual closed-loop strategy to ensure accurate spray volume control and maximize droplet deposition control.

#### 2.2.1. Development of the Mathematical Model for Precision Pesticide Application System

(1) Study of the Relationship Between Application Flow Rate, Pressure, and Solenoid Valve Input Voltage

This study precisely measured the spraying system’s pressure and flow characteristics to ensure its stability. Considering that the solenoid valve’s maximum operating voltage is 24 V, the tests were conducted within a voltage range of 0 to 24 V to accurately detect the flow rate and pressure output under different voltages.

**Figure 6:**
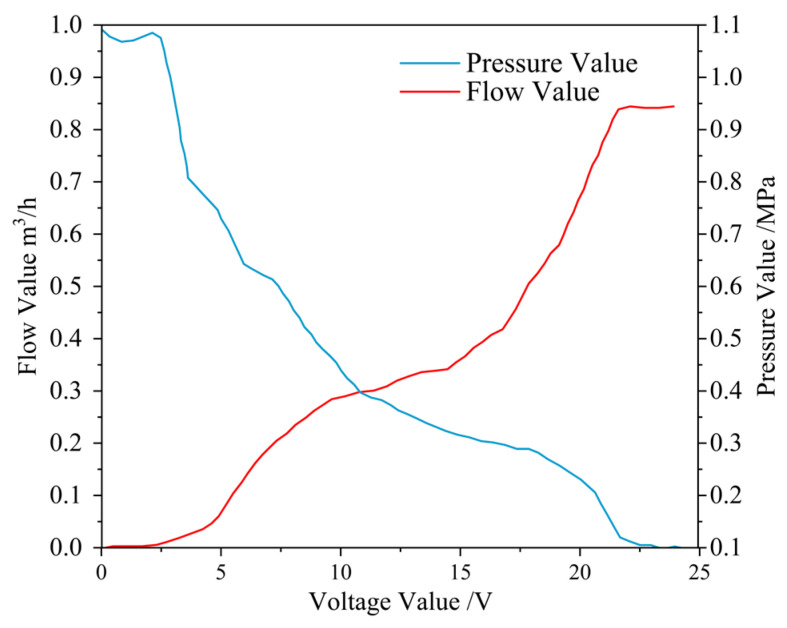
Study of the relationship between input voltage, flow rate, and pressure.

First, the relationship between the spraying flow rate and the input voltage of the solenoid valve was investigated. Under specific conditions, the spraying flow rate mainly depends on the opening degree of the flow control solenoid valve, which corresponds to the input voltage. When the input voltage *U* ≤ 2.4 V, the flow rate is close to zero; when 3.6 V ≤ *U* ≤ 21.6 V, the application flow rate increases as the solenoid valve opening increases; when *U* ≥ 22.8 V, the flow rate tends to stabilize, indicating that the solenoid valve has entered the control dead zone, as shown in Figure 6 (red). Therefore, it can be determined that the effective control range of the flow solenoid valve is 3.6 V to 21.6 V. The relationship between the application flow rate and input voltage is given by Equation (8). (Note: The relevant model was developed based on the precision spraying system and a specific type of solenoid valve used in this study under particular experimental conditions. The equipment’s structural design and dynamic response characteristics influence the associated parameters, thus exhibiting a certain degree of specificity.)


(8) $$Q=0.00028U^{3}-0.00925U^{2}+0.11744U-0.27473$$

Next, the relationship between the application pressure and the solenoid valve input voltage was investigated. The application pressure remains relatively constant when *U* ≤ 2.4 V or *U* ≥ 22.8 V. When 3.6 V ≤ *U* ≤ 21.6 V, the application pressure decreases as the solenoid valve opening increases, as shown in Figure 6 (blue). The relationship between the application pressure and input voltage is expressed by Equation (9).


(9) $$P=0.0015U^{2}-0.0724U+1.0427$$

(2) Modeling of the Pesticide Application Control System

**Figure 7:**
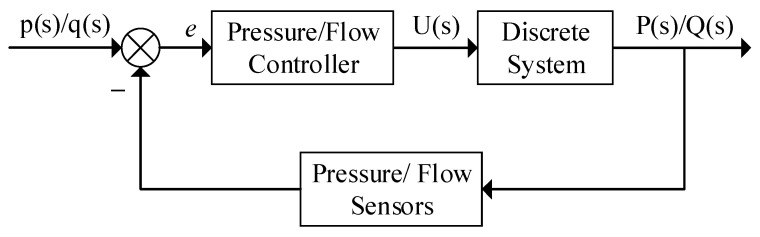
Pressure/flow control model.

In the pesticide application system, solenoid valve opening adjustment is key to precisely controlling flow rate and pressure. As shown in Figure 7, the system adopts a single closed-loop control strategy, where the flow/pressure sensor continuously monitors the pipeline parameters. Based on the monitored data, the controller outputs a voltage *U*(*s*) to adjust the solenoid valve opening, ensuring precise control of flow rate and pressure. This paper constructs the corresponding mathematical model by analyzing the relationship between flow rate, pressure, and solenoid valve input voltage.

The discretized pressure and flow models of the pesticide application system, derived from Equations (8) and (9), are shown in Equations (10) and (11).


(10) $$P(k)=0.0015U^{2}(k)-0.0724U(k)+1.0427$$

(11) $$Q(k)=0.00028U^{3}(k)-0.00925U^{2}(k)+0.11744U(k)-0.27473$$

#### 2.2.2. Design of a Fuzzy Adaptive Control Algorithm Based on ESO

**Figure 8:**
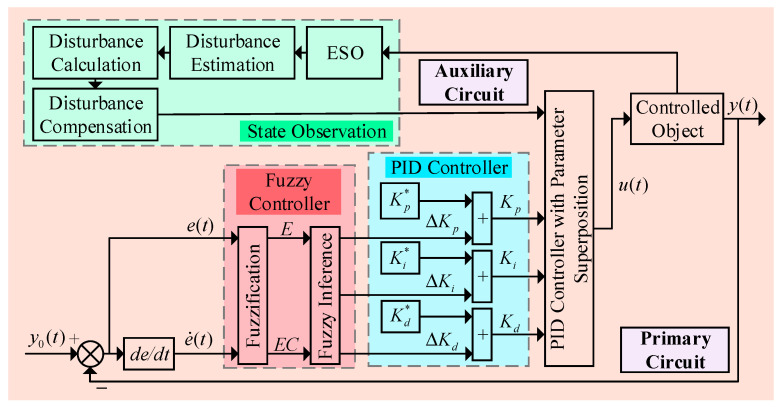
Control structure of the ESO-based fuzzy adaptive controller.

The control structure diagram of the ESO-based fuzzy adaptive controller is shown in Figure 8.

The proportional *K_p_*, integral *K_i_*, and derivative *K_d_* coefficients of the PID controller are composed of two parts: one part is the preset original PID [23] parameters K_p*_, K_i*_, and K_d*_, and the other part is the adjustment values Δ*K_p_*, Δ*K_i_*, and Δ*K_d_* calculated by the fuzzy control algorithm [24] based on real-time feedback signals. These two components are combined to form the actual PID controller parameters in this study, enabling the dynamic self-tuning of the parameters in the fuzzy adaptive PID controller. The basic equation for self-tuning is given in Equation (12).


(12) $$\left\{\begin{array}{l} K_{p}=K_{p}^{*}+\Delta K_{p}\\ K_{i}=K_{i}^{*}+\Delta K_{i}\\ K_{d}=K_{d}^{*}+\Delta K_{d} \end{array}\right.$$

(1) Establishment of the State Observer for the Precision Pesticide Application Control System

The ESO, or extended state observer, is formulated as shown in Equation (13):

(13) $$\left\{\begin{array}{l} e_{1}(t)=z_{1}(t)-y(t)\\ fe=fal(e_{1},\beta_{1},\alpha ),fe^{\prime }=fal(e_{1},\beta_{2},\alpha )\\ z_{1}(t)=z_{1}(t-1)+h\times [z_{2}(t-1)-a\times e_{1}(t-1)]\\ z_{2}(t)=z_{2}(t-1)+h\times [(z_{3}(t-1)-b\times u)-\lambda \times fe]\\ z_{3}(t)=z_{3}(t-1)-h\times c\times fe^{\prime } \end{array}\right.$$

where *y*(*t*) represents the current measurement, $z_{1}(t)$ represents the current calculated estimate, $e_{1}(t)$ denotes the difference between the estimate and the measurement, $z_{2}(t)$ is the derivative of the estimated value calculated by the ESO, $z_{3}(t)$ is the total disturbance value observed by the ESO, *h* is the sampling period, and *λ* is the gain coefficient. The nonlinear function plays fal(e,β,α) a decisive role in the performance of the observer. α is the filtering factor, and β is the nonlinear factor, typically taken as empirical values of $\beta_{1}=0.5$, $\beta_{2}=0.25$. fal(e,β,α) the function is shown in Equation (14).


(14) $$fal(e,\beta ,\alpha )=\left\{\begin{array}{l} {\left|e\right|}^{\beta }sign(e),\left|e\right|>\alpha \\ e/\alpha^{1-\beta },\left|e\right|\leq \alpha \end{array}\right.$$

The coefficients *a*, *b*, and *c* of the ESO directly affect the observer’s performance. Therefore, it is necessary to tune the values of *a*, *b*, and *c* in this study. To simplify the complexity of parameter tuning, the ESO is transformed into a linear observer form:


(15) $$\left\{\begin{array}{l} e_{1}(t)=z_{1}(t)-y(t)\\ z_{1}(t)=z_{1}(t-1)-a\times e_{1}(t-1)\\ z_{2}(t)=z_{2}(t-1)-b\times fe+f(e,e^{\prime })+b\times u\\ {\left(dz/dt\right)}_{Q3}=-h\times c\times fe^{\prime } \end{array}\right.$$

The characteristic equation obtained by applying the Laplace transform [25] to Equation (15) is as follows:


(16) $$s^{3}+as^{2}+bs+c=0$$

Taking the flow model as an example, as shown in Section 2.2.1, the flow model established in this study is a third-order model. From the characteristic equation ${\left(s+w_{c}\right)}^{3}=0$ of the third-order extended state observer, the following can be obtained:


(17) $$a=3\times w_{c},\;b=3\times w_{c}^{2},\;c=w_{c}^{3}$$

**Figure 9:**
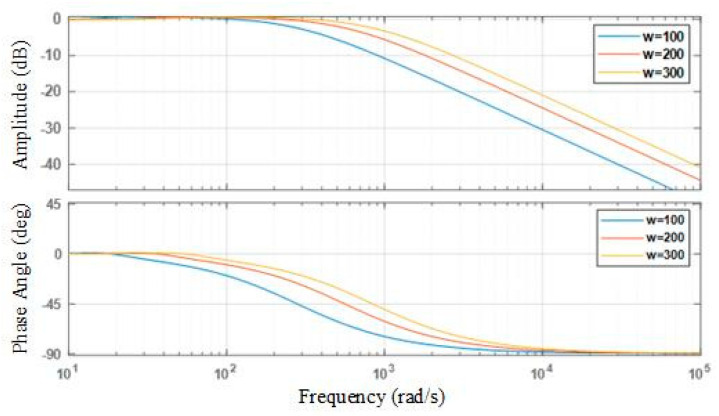
Comparison of frequency domain characteristics curves corresponding to different *w_c_* values.

In Equation (17), *w_c_* is the system bandwidth, with values of *w_c_* = 100, 200, and 300; the frequency-domain characteristic curves are shown in Figure 9. As *w_c_* increases, the curve shifts to the right, the phase lag decreases, the ESO estimation error convergence rate increases, and the system’s dynamic performance improves.

In the frequency-domain analysis, the optimal values of the extended state observer (ESO) parameters are determined by identifying the frequency bands where the control effect is most prominent, thereby providing a basis for tuning the ESO parameters. The tuning results are shown in Table 2.

After completing the disturbance observation and calculation, a disturbance compensation module for the pressure and flow controllers is designed. Based on the ESO observation results, this module compensates for the total system disturbance in real-time, thereby improving the system’s resistance to disturbances [26]. The form of disturbance compensation is shown below:


(18) $$u=u_{0}-d(t)$$

(19) $$d(t)=\frac{z_{3}(t)}{\lambda }$$

In Equation (19), *u* represents the actual output value of the controller, *u*_0_ is the theoretical output value of the controller, and *d*(*t*) is the system disturbance compensation.

(2) Definition of Input and Output Fuzzy Sets

To achieve fuzzy adaptive PID control, the input variables (error e in pesticide flow rate/pressure and the rate of change in the error *de*/*dt*) and the output variables Δ*K_p_*, Δ*K_i_*, and Δ*K_d_* are each divided into seven fuzzy sets. These sets are described using linguistic variables: “NB (Negative Big), NM (Negative Medium), NS (Negative Small), ZE (Zero), PS (Positive Small), PM (Positive Medium), PB (Positive Big).” Considering practical engineering requirements, the fuzzy domain for both the error and the rate of change in the error is defined as [−3, 3], with values exceeding this range being treated as boundary values to enhance system robustness.


(20) $$E=\left\{-3,-2,-1,0,1,2,3\right\}$$

In Equation (20), *E* represents the input error’s fuzzy domain and the error’s rate of change.

(3) Establishment of Fuzzy Control Rules

Based on expert experience, a fuzzy rule base is established. In this study, fuzzy control rule tables for Δ*K_p_*, Δ*K_i_*, and Δ*K_d_* are formulated, as shown in Table 3. The fuzzy control rules are derived from the expertise of specialists in agricultural spraying control rather than being automatically generated through data-driven machine learning methods.

(4) Establishment of Fuzzy Rule Lookup Table

Based on fuzzy inference, the fuzzy output is defuzzified using the centroid method to obtain precise output values, which are then rounded to one decimal place. The control parameter lookup tables for Δ*K_p_*, Δ*K_i_*, and Δ*K_d_* are constructed, as shown in Table 4. However, before combining these values with the original PID parameters, these adjustment values must be multiplied by the corresponding scaling factors *K_up_*, *K_ui_*, and *K_ud_*.

The sampling bias and the rate of bias change are first processed through fuzzy handling, and new control parameters are determined based on fuzzy control rules. Then, the ESO (extended state observer) is used to detect and compute system disturbances, generating a compensation signal. Finally, after parameter adjustment, the controller combines the disturbance compensation to compute the output, which is the system control quantity.

In this paper, the incremental PID algorithm [27] is chosen, and its equation is as follows:


(21) $$\Delta u(t)=u(t)-u(t-1)=K_{p}\times [\;e(t)-e(t-1)]+K_{i}\times e(t)+K_{d}\times [\;e(t)-2\times e(t-1)+e(t-2)]-d(t)$$

In the Equation, *e*(*t*), *e*(*t* − 1), and *e*(*t* − 2) represent the errors between the actual and theoretical values of the system at time *t*, *t* − 1, and *t* − 2, respectively. *K_p_*, *K_i_*, and *K_d_* are the PID parameters after self-tuning. *d*(*t*) is the system disturbance compensation, while *u*(*t*) and *u*(*t* − 1) represent the system output at time *t* and *t* − 1, respectively. The term Δ*u*(*t*) represents the increment of the output at time *t*.

### 2.3. Experimental Setup and Conditions

#### 2.3.1. Construction of the Experimental Setup and Cost Analysis

**Figure 10:**
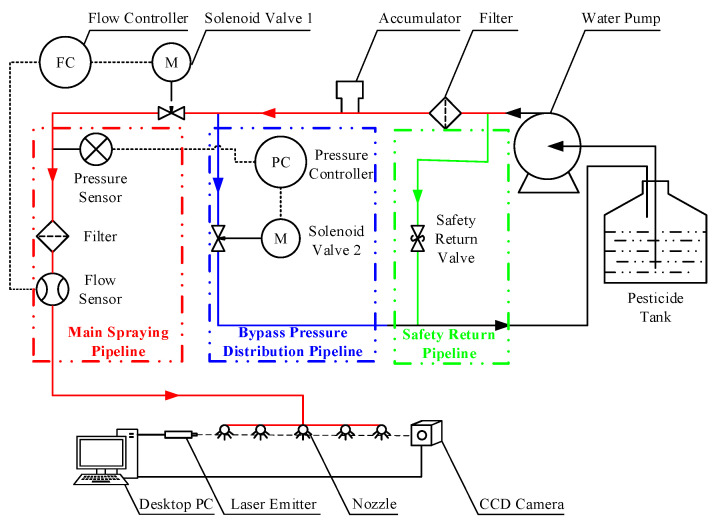
Spraying pipeline structure of the precision spraying control system.

**Figure 11:**
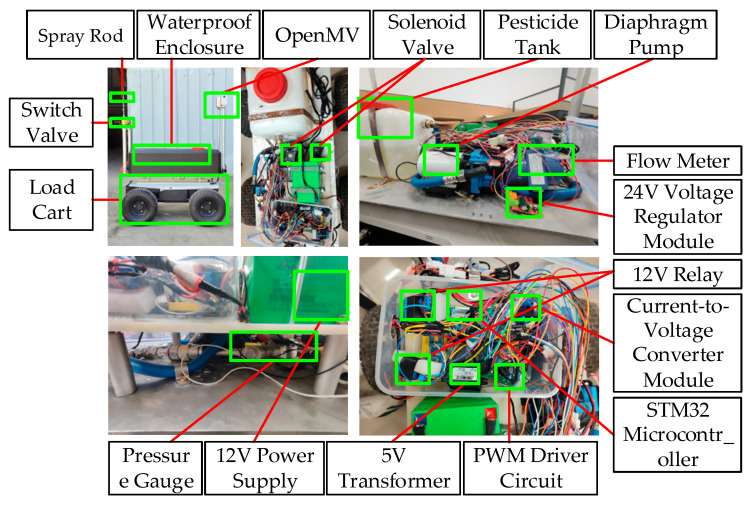
Physical diagram of the precision spraying system.

The precision spraying control system designed in this paper consists of a pesticide tank, pump, pressure gauge, filter, accumulator, solenoid valve, flow sensor, and other components (Chongqing, China). The spraying pipeline includes the main spray pipeline, bypass pressure regulation pipeline, and safety return pipeline. During spraying, the diaphragm pump draws the chemical solution from the tank and injects it into the main spray pipeline. If the main pipeline becomes blocked, the solution is redirected to the tank through the safety return pipeline. The spray pipeline is divided into the main pipeline, which controls the flow, and the bypass pressure regulation pipeline, which controls the pressure. The bypass outlet is connected to the pesticide tank, while the main pipeline outlet is connected to the nozzle, with pressure sensors and flow sensors connected in series. The system’s spraying pipeline structure is shown in Figure 10, and the physical system of the precision spraying system is shown in Figure 11.

In this study, the total cost of the machine vision-based system is approximately 1767 CNY. The system primarily consists of an industrial camera (manufacturer Hikvision, version MV-CA013-20UM, Hangzhou, China) (799 CNY), an STM32 microcontroller (manufacturer STMicroelectronics, version STM32F103ZET6, Chongqing, China) combined with an OpenMV 4Plus module (manufacturer OpenMV, version 4.6.20, Chongqing, China) (618 CNY), and a CKD (manufacturer CKD Corporation, version ADK11-15A-4E, Nagoya, Japan) solenoid valve (350 CNY).

By contrast, a representative LiDAR-based system costs approximately 31,850 CNY, including a Velodyne LiDAR sensor (manufacturer Velodyne Lidar, Inc, version VLP-16, San Jose, CA, USA) (28,300 CNY), an NVIDIA processor (manufacturer NVIDIA Corporation, version Jetson Xavier NX, Santa Clara, CA, USA) (3200 CNY), and the same model of solenoid valve (CKD, 350 CNY).

These figures indicate that, compared to the LiDAR-based solution, the machine vision system offers a significant cost advantage for large-scale orchard applications.

#### 2.3.2. Experimental Condition Configuration

(1) Indoor Experimental Scenario Layout

**Figure 12:**
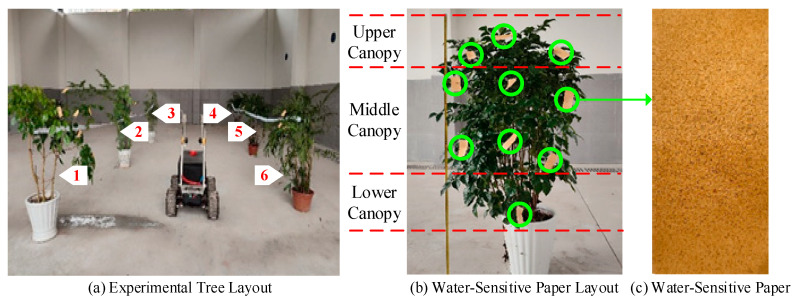
Experimental setup.

To evaluate the optimal spraying pressure and assess the performance of the control algorithm, a series of laboratory-based precision spraying experiments were conducted under controlled conditions, as illustrated in Figure 12. Due to spatial limitations, three test groups were performed indoors. Each group included six trees with varying canopy sizes, densities, and structural characteristics, resulting in 18 trees. The trees were arranged in two staggered rows, with three trees per row, spaced 3.4 m apart between rows and 2.5 m apart within each row. The precision spraying system traveled along the centerline between the two rows, maintaining an approximate nozzle-to-canopy distance of 1.3 m, as shown in Figure 12a. Water-sensitive papers were affixed to different regions of the tree canopy to capture droplet distribution, as depicted in Figure 12b. After spraying, the water-sensitive papers were air-dried, collected, and subsequently analyzed to evaluate the spraying characteristics, as shown in Figure 12c.

(2) Field Experimental Scenario Layout

**Figure 13:**
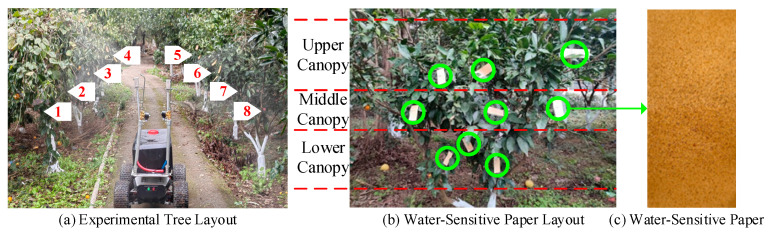
Field experiment setup.

Due to field space constraints, three field tests were conducted, each involving eight citrus trees with an average height of 1.5 m, totaling 24 trees. The trees were spaced 3 m apart between rows. Four spraying strategies were compared: continuous spraying, PID control, fuzzy adaptive control, and ESO-based fuzzy adaptive control. To evaluate the spraying effectiveness, three pieces of water-sensitive paper were randomly placed on each tree’s upper, middle, and lower canopy layers to collect droplets, as illustrated in Figure 13.

## 3. Results and Discussion

### 3.1. Simulation Based on the ESO-Fuzzy Adaptive Control Algorithm

#### 3.1.1. Simulation Validation of the ESO

**Figure 14:**
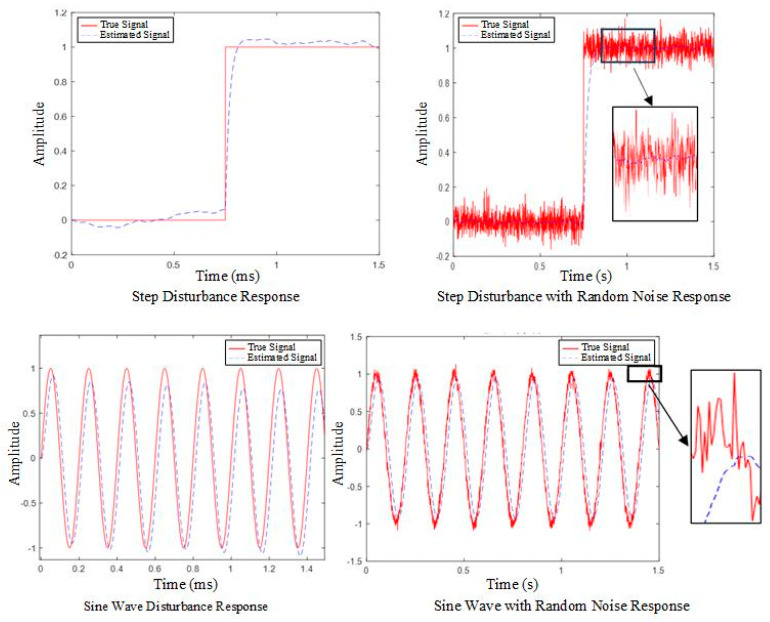
ESO effectiveness validation results.

Simulation experiments were conducted using MATLAB to verify the effectiveness of the ESO. The experiments were divided into four groups: the first group was a step response disturbance, the second group was a step response with superimposed random noise, the third group was a sinusoidal function disturbance, and the fourth group was a sinusoidal function with superimposed random noise. The simulation results are shown in Figure 14.

#### 3.1.2. Simulation of Precision Spraying Control System

(1) Simulation of Precision Spraying Pressure Closed-Loop Control System

**Figure 15:**
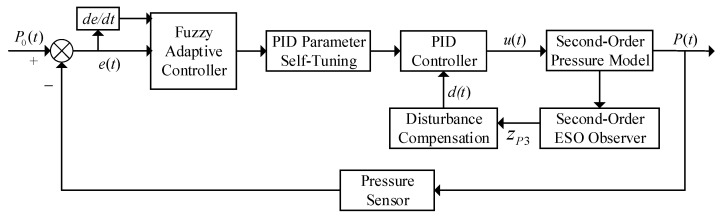
Precision spraying pressure closed-loop controller.

The pressure closed-loop controller designed in this paper is shown in Figure 15. A second-order controller structure is adopted for the second-order pressure model.

In the pressure closed-loop controller designed in this paper, the pressure sensor monitors the system’s spraying pressure *P*(*t*) in real time. By comparing the actual pressure value *P*(*t*) with the preset pressure value *P*_0_(*t*), the pressure error *e*(*t*) and its rate of change *de*/*dt* are calculated.

Disturbances such as equipment vibration and actuator deviations can occur in practical spraying operations. In this paper, a disturbance signal is introduced to compare the performance of pressure controllers using PID control, fuzzy adaptive control, and ESO-based fuzzy adaptive control, in order to evaluate their disturbance rejection capabilities.

**Figure 16:**
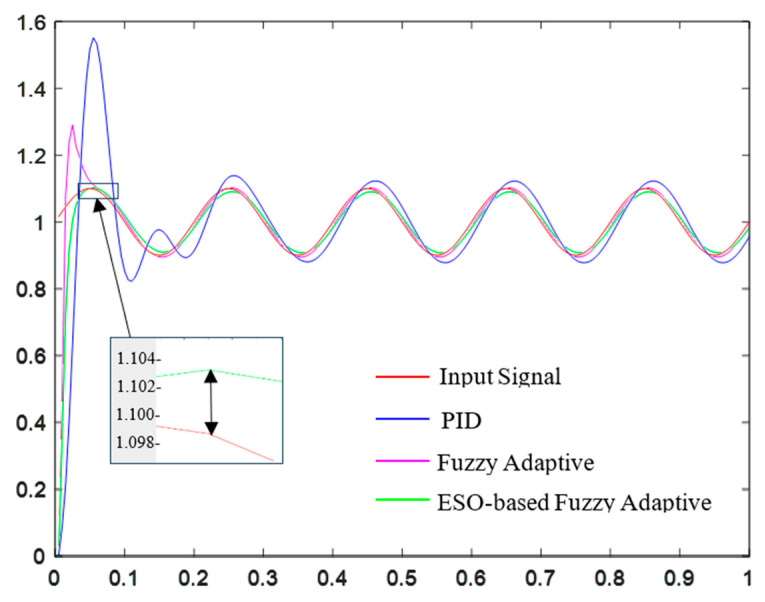
Analysis of antidisturbance capability of three pressure controllers.

As shown in Figure 16, after introducing disturbances, the PID pressure controller has a settling time of 0.34 s and a maximum overshoot of 43.64%; the fuzzy adaptive controller has a settling time of 0.15 s and a maximum overshoot of 22.73%; the ESO-based fuzzy adaptive controller has a settling time of 0.165 s and a maximum overshoot of 1.8%. The simulation results demonstrate that the ESO-based fuzzy adaptive controller performs excellently in terms of response speed and overshoot, exhibiting strong disturbance rejection capability and thereby enhancing the stability and reliability of the precision spraying system.

(2) Simulation of Precision Spraying Flow Closed-Loop Control System

**Figure 17:**
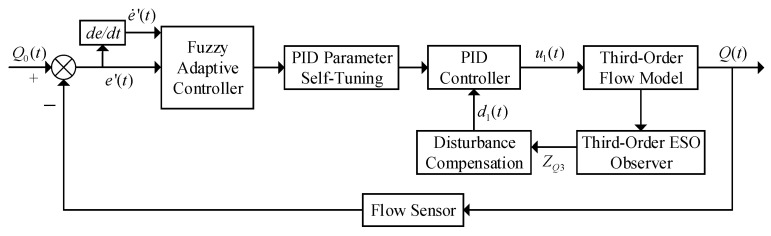
Structure diagram of precision spraying flow closed-loop controller.

The flow closed-loop controller designed in this paper is shown in Figure 17. For the third-order flow model, a third-order controller structure is adopted.

**Figure 18:**
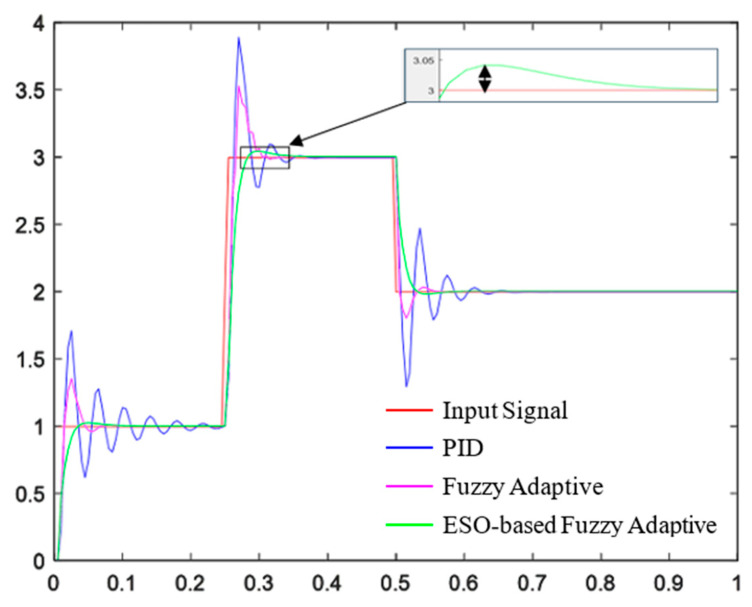
Tracking performance analysis of three flow controllers.

In practical spraying operations, differences in canopy leaf wall area and density require dynamic adjustment of the spraying amount. Therefore, during the simulation, the input signal considers changes in the spraying amount. The tracking performance of different controllers is compared. As shown in Figure 18, the settling times for the PID controller are 0.11 s, 0.38 s, and 0.67 s, with a maximum overshoot of 65%. The settling times for the fuzzy adaptive controller are 0.075 s, 0.325 s, and 0.56 s, with a maximum overshoot of 35.6%. The settling times for the ESO-based fuzzy adaptive controller are 0.075 s, 0.325 s, and 0.56 s, with a maximum overshoot of 1.6%. The simulation results show that the ESO-based fuzzy adaptive controller exhibits faster response times and smaller overshoots in flow control, improving the system’s control performance and stability.

### 3.2. Indoor Experiment and Result Analysis

#### 3.2.1. Spraying Pressure Measurement

The pipeline pressure is adjusted by maintaining a constant flow to find the optimal spraying pressure. Expert experience suggests that within the pressure range of 0.2 MPa to 0.8 MPa, the droplet size variation is moderate, which can reduce droplet roll-off, drift, and rebound. To determine the optimal pressure value, pre-experiments were sprayed using landscape trees A, B, and C, with all leaves covered with water-sensitive paper. The vertical distance between the nozzle and the trunk was set to 0.8 m, and a fixed-point spraying method was used to record the droplet deposition effects under different pressures.

**Figure 19:**
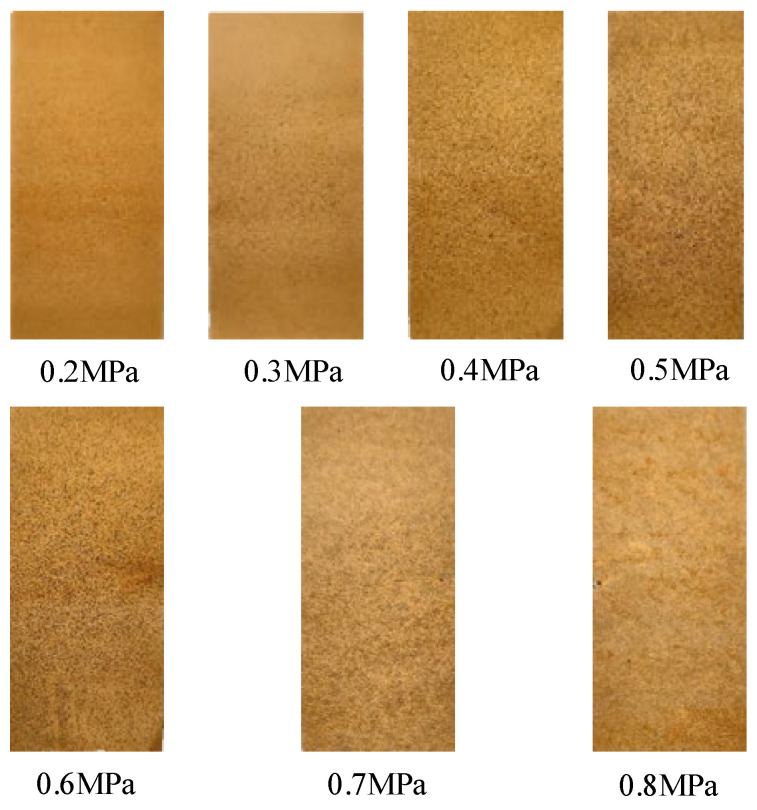
Schematic of Water-Sensitive Paper Under Pressures of 0.2–0.8 MPa.

**Figure 20:**
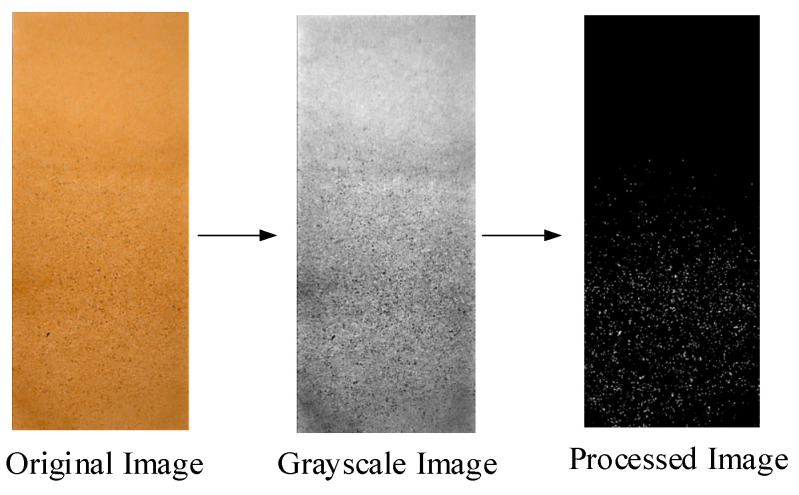
Process of water-sensitive paper treatment.

After the spraying test, the dried water-sensitive paper (as shown in Figure 19) was placed in labeled self-sealing bags, and the images of the water-sensitive paper were processed using ImageJ software (version 1.53k) (as shown in Figure 20). The droplet coverage, number of deposition points, and deposition point density were analyzed.

The number of deposition points refers to the number of droplets, where the droplets spread on the water-sensitive paper to form black spots, and the number of spots is counted to determine the deposition point count. The droplet coverage rate is the ratio of the droplet area to the area of the water-sensitive paper, reflecting the deposition level. The calculation equation is shown in Equation (22).


(22) $$C=\frac{A_{S}}{A_{P}}\times 100\%$$

In the equation, *C* represents the droplet coverage rate, in percentage (%); *A_S_* is the total droplet deposition area pixel value on the water-sensitive paper; *A_P_* is the pixel value of the water-sensitive paper area.

The droplet deposition point density indicates the number of deposition points per unit area on the collection card. The calculation equation is shown in Equation (23).


(23) $$K=\frac{N}{M}$$

In the equation, *K* represents the droplet deposition point density in droplets/cm^2^; *N* is the total number of droplets; *M* is the total area of the test paper in cm^2^.

The droplet coverage rate and droplet point deposition density for a single tree under precision spraying conditions are calculated. After repeated experiments, the average value of three trees is taken as the final result for spraying on landscape trees, as shown in Table 5.

As shown in Table 5, the spraying pressure significantly affects the droplet deposition performance in precision spraying systems. At a pressure of 0.2 MPa, the droplet deposition density was only 9.12 drops/cm^2^, with a canopy coverage of 10.54% and a deposition efficiency of 25.5%. Under such conditions, droplets tend to be larger and possess insufficient kinetic energy, resulting in low deposition density and weak adhesion to leaf surfaces, limiting the pesticide utilization efficiency. As the spraying pressure increases, both the deposition density and coverage improve significantly. When the pressure reaches 0.6 MPa, the canopy coverage peaks at 70.07%, and the deposition efficiency reaches 87%, indicating an optimal balance between the droplet atomization and canopy interaction.

However, further increasing the pressure to 0.7 MPa and 0.8 MPa leads to a decline in the deposition efficiency to 66% and 59.5%, respectively, despite relatively high coverage rates of 58.81% and 65.74%. This is mainly because excessive pressure produces overly fine droplets with reduced inertia, making them more susceptible to drift and runoff. The adhesion efficiency of droplets on leaf surfaces depends on the coupling relationship among droplet size, velocity, and leaf surface structure. Tiny droplets are more likely to coalesce due to surface tension upon contact and roll-off, increasing pesticide loss.

Therefore, the spraying pressure nonlinearly influences the droplet deposition quantity, spatial density, and retention stability. Appropriate regulation of the spray pressure ensures sufficient atomization and enhances the droplet adhesion to the fruit tree canopy, thereby improving pesticide utilization and reducing resource waste while maintaining adequate coverage [28].

#### 3.2.2. Control Algorithm Optimization

**Figure 21:**
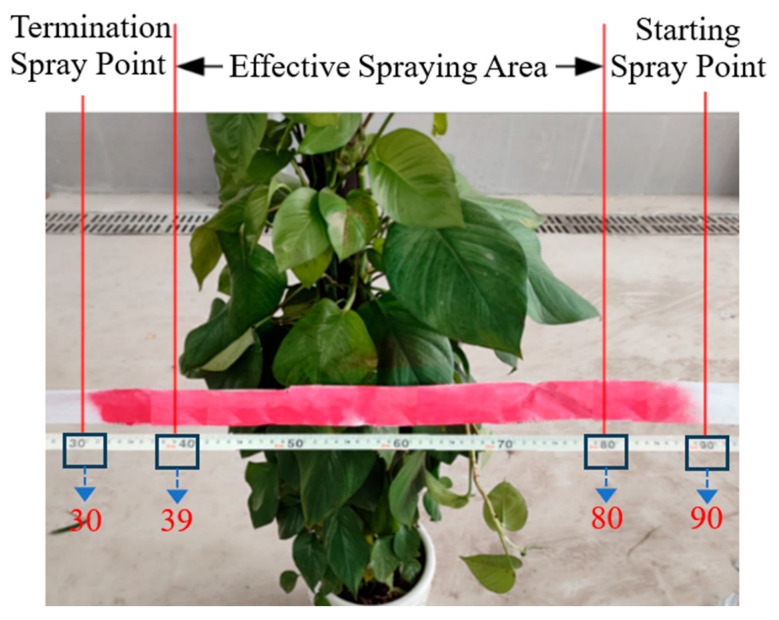
Schematic of the effective spray area.

A strip of test paper is placed in the middle of the tree canopy along a direction parallel to the ground. As the spraying device passes, the test paper reflects the maximum spraying distance when the device passes over the tree. By comparing the canopy width of the tree, the performance of the spraying control algorithm is evaluated. The sprayed area of the test paper is stained with the chemical solution, while the unsprayed area remains white, as shown in Figure 21.

The unmanned plant protection machine travels along the midline of the two rows at a speed of 0.4 m/s, recording the variable spraying effects based on PID control, fuzzy control, and ESO-based fuzzy adaptive control, as shown in Table 6. The spraying effect is the average of three spraying operations for each control algorithm.

This paper evaluates the spraying effect through the average deposition rate, deposition density, and spraying accuracy. The average deposition rate is the ratio of the actual total spraying amount to the deposition amount. The deposition density is measured by counting the droplets on the water-sensitive paper, reflecting the number of droplets per unit area on the tree. The spraying accuracy is the ratio of the actual spraying amount to the set spraying amount, which is determined by the spraying amount calculation model.

The actual spraying amount is the total amount of liquid sprayed by the nozzle, calculated by multiplying the flow values detected by the flow sensor at each period via the total spraying duration. The deposition amount refers to the amount of liquid that finally adheres to the tree leaves. The calculation equation is as follows:


(24) $$L=\frac{N\times \frac{4}{3}\pi r^{3}}{W}\times \frac{S_{a}}{S_{b}}$$

(25) $$\sigma =\frac{L}{V_{S}}\times 100\%$$

In Equations (24) and (25), *L* represents the deposition amount on the tree, *N* is the total number of droplets on the water-sensitive paper, r is the median droplet radius measured on the water-sensitive paper using ImageJ software, *W* is the number of water-sensitive pieces of paper used on the tree, *S_a_* is the area of the tree canopy, *S_b_* is the area of a single piece of water-sensitive paper, *σ* is the droplet deposition rate, and vs. is the actual spraying amount on the tree.

The spraying accuracy is one of the key indicators for evaluating the performance of a spraying system. It is commonly used to quantify the deviation between the actual and the target spray volumes. The calculation is given as follows:


(26) $$Spray\;Accuracy=1-\frac{\left|Q_{set}-Q_{act}\right|}{Q_{set}}$$

In Equation (26), *Q_set_* denotes the total target spray volume, *Q_act_* denotes the actual spray volume, and | ∙ |represents the absolute value operation.

The ESO-based fuzzy adaptive control algorithm demonstrates excellent performance in droplet deposition, significantly improving both spraying accuracy and average deposition rate. Experiments show that its spraying accuracy is 64.11% higher than continuous spraying, 29.03% higher than the PID algorithm, and 16.13% higher than the fuzzy adaptive algorithm. The average deposition rate is 60.98% higher than continuous spraying, 26.19% higher than the PID algorithm, and 13.98% higher than the fuzzy adaptive algorithm.

### 3.3. Field Experiments and Result Analysis

#### 3.3.1. Field Experiment on Machine Vision-Based Canopy Recognition

To evaluate the accuracy of the precision spraying dosage model based on machine vision recognition of canopy structural parameters, the plant protection spraying system was tested under various environmental conditions, including overcast, rainy, and sunny weather, as well as in the morning, at noon, and in the evening. Under each condition, the canopy leaf wall area was identified and calculated. The resulting variations in leaf wall area measurements under different weather scenarios are summarized in Table 7. The reference values for leaf wall area errors were obtained through manual geometric segmentation, which served as the ground truth.

It was observed that both excessive brightness and insufficient lighting negatively impacted the accuracy of the leaf wall area estimation based on machine vision. In light of this, researchers recommend conducting field spraying operations on overcast days to mitigate the effect of lighting variability on computational precision.

Considering the influence of lighting conditions on the estimation accuracy and expert recommendations, the optimal spraying periods are from 9:00 to noon and 3:00 to 6:00 p.m. These periods offer moderate lighting and suitable ambient temperatures, which help maximize the performance of the machine vision-based leaf wall area estimation method, thereby ensuring both accuracy and operational efficiency.

#### 3.3.2. Field Experiment on Precision Spraying

To verify the effectiveness of the ESO-based fuzzy adaptive variable spraying control algorithm in pesticide savings, the experiment compared the pesticide usage under different control methods. The experiment was conducted in the same orchard, and the test results are shown in Table 8.

The experimental results show that the average spraying accuracy with the ESO-based fuzzy adaptive control algorithm is 91.38%. Compared to continuous spraying, this algorithm reduces the spraying volume by 59.73%; compared to PID control, it reduces the spraying volume by 30.24%; compared to traditional fuzzy control, it reduces the spraying volume by 19.19%. Additionally, the deposition rate increases by 61.42% compared to continuous spraying, by 26.8% compared to PID control, and by 19.54% compared to traditional fuzzy control. This indicates that the algorithm ensures effective spraying and improves pesticide utilization, further reducing pesticide usage.

### 3.4. Discussion of Experimental Results

(1) Limitations and Solutions for Orchard Canopy Detection

Canopy detection is crucial for determining the spraying accuracy in orchard precision spraying systems. Although the GrabCut-algorithm-based canopy segmentation performs excellently in standard environments, its segmentation accuracy is limited in strong sunlight or overlapping leaves. Strong sunlight affects the reflective properties of the leaves, making it difficult to distinguish between the background and the foreground, while overlapping leaves prevent GrabCut from accurately identifying the independent contours of each leaf, thus impacting the calculation of the canopy area.

To address this issue, future research may consider introducing lightweight deep learning methods, such as MobileNet and ShuffleNet, which optimize network performance. Compared to traditional algorithms, deep learning methods are more robust when handling complex lighting changes and overlapping leaves. They can reduce the computational burden while maintaining high segmentation accuracy, thus meeting real-time processing requirements. Therefore, introducing deep learning techniques is expected to significantly enhance the system’s adaptability in complex environments, particularly under challenging conditions such as strong sunlight and leaf overlap.

(2) Comparison between Simulation and Experimental Results

The fuzzy adaptive controller based on ESO designed in this study demonstrates good dynamic performance in simulation, with an overshoot of only 1.6% and a stable response. However, the indoor and field experimental results show spraying accuracies of 95.56% and 91.38%, respectively. This discrepancy primarily arises from the simulation environment not fully encompassing engineering factors such as sensor noise, sampling delay, solenoid valve response lag, and the inertia of the pump and pipeline system, all of which are present in the indoor experiments and lead to a slight degradation in control performance. Moreover, the complexity of the field environment, including factors like wind speed, wind direction, tree structure, and terrain, also challenges the system’s stability.

(3) Comparison of Precision Spraying Effects with Existing Studies

Compared with previous research, the proposed system shows notable improvements in pesticide saving rate and control accuracy. For instance, Guo et al. employed a YOLOv8n-DT-based UAV spraying system, achieving a 15.28% pesticide reduction compared to traditional methods [29]; Hočevar et al. developed an RGB image-based profile sprayer with an average saving of 23.53% [30]; Nan et al. utilized a contour-based variable-rate spraying approach with ultrasonic sensing, achieving a 32.1% reduction [31]. In contrast, the system proposed in this study achieved a 59.73% pesticide saving in real field conditions, outperforming the aforementioned methods.

Moreover, the control system’s high efficiency is also attributed to its ESO, which can estimate system disturbances in real-time and adjust control parameters through fuzzy logic. This allows the system to maintain high spraying accuracy in complex environments and exhibit good dynamic response capabilities in the presence of disturbances.

## 4. Conclusions

The precision spraying system proposed in this study demonstrates excellent adaptability across different tree species. Through parameter-adaptive mechanisms and optimization algorithms, the system can effectively handle the diversity of fruit trees with different canopy structures, such as apples, pears, and grapes, dynamically adjusting spraying parameters based on the growth characteristics and pest control needs of each species, achieving precise spraying in diverse orchard conditions. By employing precise image recognition and segmentation methods, the system can accurately extract structural and canopy features of various fruit trees, enabling on-demand spraying, improving resource utilization efficiency, and promoting the achievement of green pest control objectives.

The system employs a highly modular and standardized interface design at the hardware and software architecture level to achieve efficient integration with automated operation platforms. Each functional unit interacts and collaborates through a unified protocol, allowing the system to be directly integrated into existing uncrewed spray vehicles, automated tractors, and other mainstream automation equipment without significantly modifying the platform’s underlying structure. This architecture reduces the difficulty of platform adaptation between heterogeneous systems and provides a solid foundation for future seamless integration with new intelligent agricultural machinery and system function expansion.

Future work will focus on three areas: (1) enhancing the system’s robustness to extreme lighting and complex backgrounds by incorporating multi-modal sensing and adaptive image enhancement methods; (2) expanding the model’s online learning and self-calibration capabilities to improve the environmental adaptability and timeliness of spraying decisions; and (3) integrating task planning, collaborative control, and edge computing to push the system toward multi-machine collaboration and autonomous operation in intelligent group spraying.

This study provides a low-cost, highly reliable, and scalable solution for intelligent precision spraying in complex orchard environments. It offers significant support for applying green pest control technologies in agriculture.

## Figures and Tables

**Table 1:** Comparison of segmentation performance between GrabCut and thresholding methods.

Method	Iterations	Data Term Weight	Smoothness Term Weight	IoU	Precision	Recall
Thresholding	N/A	N/A	N/A	0.62	0.65	0.60
GrabCut	20	0.7	0.35	0.85	0.88	0.83

**Table 2:** ESO coefficient values.

Parameters	Value Range	Tuned Values
*a*	50–230	100
*b*	150–250	200
*c*	250–750	500

**Table 3:** Fuzzy control rules for Δ*K_p_*, Δ*K_i_*, and Δ*K_d_*.

*e*(*t*)	*de*/*dt*						
	NB	NM	NS	ZE	PS	PM	PB
NB	PB/NB/PS	PB/NB/NS	PM/NM/NB	PM/NM/NB	PS/NS/NB	ZE/ZE/NM	ZE/ZE/PS
NM	PB/NB/PS	PB/NB/NS	PM/NM/NB	PS/NS/NM	PS/NS/NM	ZE/ZE/NS	NS/ZE/ZE
NS	PM/NB/ZE	PM/NM/NS	PM/NS/NM	PS/NS/NM	ZE/ZE/NS	NS/PS/NS	NS/PS/ZE
ZE	PM/NB/ZE	PM/NM/NS	PS/NS/NS	ZE/ZE/NS	NS/PS/NS	NM/PM/NS	NM/PM/ZE
PS	PS/NM/ZE	PS/NS/PS	ZE/ZE/ZE	NS/PS/ZE	NS/PS/ZE	NM/PM/ZE	NM/PB/NS
PM	PS/ZE/PB	ZE/ZE/NS	NS/PS/PS	NM/PS/PS	NM/PM/PS	NM/PB/PS	NB/PB/PB
PB	ZE/ZE/PB	ZE/ZE/PM	NM/PS/PM	NM/PM/PM	NM/PM/PS	NB/PB/PS	NB/PB/PS

**Table 4:** Fuzzy control rule lookup table for Δ*K_p_*, Δ*K_i_*, and Δ*K_d_*.

*e*(*t*)	*de*/*dt*						
	−3/	−2	−1	0	1	2	3
−3	−0.3/−0.3/0.1	−0.3/−0.3/0.1	−0.3/−0.3/0.0	−0.2/−0.2/0.0	−0.2/−0.2/0.1	0.0/0.0/0.3	0.0/0.0/0.3
−2	−0.3/−0.3/−0.1	−0.3/−0.3/−0.1	−0.2/−0.2/−0.1	−0.2/−0.2/−0.1	−0.1/−0.1/0.0	0.0/0.0/−0.1	0.0/0.0/0.2
−1	−0.2/−0.2/−0.3	−0.2/−0.2/−0.3	−0.1/−0.1/−0.2	−0.1/−0.1/−0.1	0.0/0.0/0.0	0.1/0.1/0.1	0.1/0.1/0.2
0	—/−0.2/−0.3	—/−0.1/−0.2	—/−0.1/−0.2	—/0.0/−0.1	—/0.1/0.0	—/0.1/0.1	—/0.2/0.2
1	−0.1/−0.1/−0.3	−0.1/−0.1/−0.2	0.0/0.0/−0.1	0.1/0.1/−0.1	0.1/0.1/0.0	0.2/0.2/0.1	0.2/0.2/0.1
2	0.0/0.0/−0.2	0.0/0.0/−0.1	0.1/0.1/−0.1	0.2/0.2/−0.1	0.2/0.2/0.0	0.3/0.3/0.1	0.3/0.3/0.1
3	0.0/0.0/0.1	0.0/0.0/0.0	0.1/0.1/0.0	0.2/0.2/0.0	0.3/0.3/0.0	0.3/0.3/0.3	0.3/0.3/0.3

**Table 6:** Comparison of the effects of four spraying methods.

Control Algorithm	Set Spray Volume (m^3^)	Actual Spray Volume (m^3^)	Droplet Deposition Volume (m^3^)	Deposition Density (Drops/cm^2^)	Spray Accuracy	Average Deposition Rate
Continuous Spraying	2.48	4.18	1.33	53.93	31.45%	30.16%
PID	2.48	3.31	2.15	110.92	66.53%	64.95%
Fuzzy Control	2.48	1.97	1.52	118.54	79.43%	77.16%
ESO-Based Fuzzy Adaptive Control	2.48	2.37	2.16	151.69	95.56%	91.14%

**Table 7:** Measurement results of canopy leaf wall area under different weather conditions.

Tree ID	Environment	Leaf Wall Area (cm^2^)	Leaf Wall Area Error	Environment	Leaf Wall Area (cm^2^)	Leaf Wall Area Error
A	Overcast	14,779.37	7.18%	Foggy	8878.84	35.61%
A	Light Rain	9981.29	27.62%	Clear Noon	15,779.37	14.43%
A	Clear Evening	16,112.45	16.84%	Clear Morning	15,891.64	15.24%
B	Overcast	16,312.24	11.51%	Foggy	10,437.34	28.65%
B	Light Rain	10,897.45	25.51%	Clear Noon	16,990.83	16.15%
B	Clear Evening	17,425.36	19.12%	Clear Morning	16,645.21	13.78%
C	Overcast	17,985.74	7.49%	Foggy	10,784.23	35.54%
C	Light Rain	12,896.46	22.92%	Clear Noon	18,844.93	12.63%
C	Clear Evening	19,456.36	16.28%	Clear Morning	19,047.69	13.84%

**Table 8:** Comparison of the effects of four spraying methods in the field.

Method	Set Spray Volume (m^3^)	Actual Spray Volume (m^3^)	Leaf Surface Deposition Volume (m^3^)	Spray Accuracy	Deposition Rate
Continuous Spraying	3.56	5.89	1.66	34.55%	28.18%
	3.56	6.01	1.64	31.18%	27.15%
	3.56	6.08	1.71	29.21%	28.13%
PID	3.56	4.93	3.09	61.52%	62.68%
	3.56	4.94	3.03	61.24%	61.34%
	3.56	4.96	3.14	60.67%	63.31%
Fuzzy Control	3.56	2.55	1.83	71.63%	71.76%
	3.56	2.58	1.82	72.47%	70.54%
	3.56	2.58	1.85	72.47%	71.71%
ESO-Based Fuzzy Adaptive Control	3.56	3.28	2.98	92.13%	90.85%
	3.56	3.23	2.95	90.73%	91.33%
	3.56	3.25	2.94	91.29%	90.46%

**Table 5:** Spray effect measurement under different pressures.

Spray Pressure (MPa)	Average Droplet Deposit Density (Drops/cm^2^)	Droplet Coverage	Total Spray Volume (m^3^)	Total Deposit Volume (m^3^)	Droplet Deposition Rate
0.2	9.12	10.54%	0.2	0.051	25.5%
0.3	20.6	13.08%	0.2	0.064	32%
0.4	64.78	18.73%	0.2	0.088	44%
0.5	141.08	48.3%	0.2	0.112	61%
0.6	110.99	70.07%	0.2	0.174	87%
0.7	71.85	58.81%	0.2	0.122	66%
0.8	35.23	65.74%	0.2	0.119	59.5%

## Data Availability

The data will be provided upon request.
